# Comparative Untargeted Metabolic Profiling of Different Parts of *Citrus sinensis* Fruits via Liquid Chromatography–Mass Spectrometry Coupled with Multivariate Data Analyses to Unravel Authenticity

**DOI:** 10.3390/foods12030579

**Published:** 2023-01-29

**Authors:** Sherif M. Afifi, Eman M. Kabbash, Ralf G. Berger, Ulrich Krings, Tuba Esatbeyoglu

**Affiliations:** 1 Pharmacognosy Department, Faculty of Pharmacy, University of Sadat City, Sadat City 32897, Egypt; 2 Department of Food Development and Food Quality, Institute of Food Science and Human Nutrition, Gottfried Wilhelm Leibniz University Hannover, Am Kleinen Felde 30, 30167 Hannover, Germany; 3 Phytochemistry Department, National Organization for Drug Control and Research, Giza 12622, Egypt; 4 Institute of Food Chemistry, Gottfried Wilhelm Leibniz University Hannover, Callinstraße 5, 30167 Hannover, Germany

**Keywords:** albedo, flavedo, concentrate, juice, orange, metabolomics, flavonoids, coumarins

## Abstract

Differences between seven authentic samples of *Citrus sinensis* var. Valencia peel (albedo and flavedo) and juices from Spain and Uruguay, in addition to a concentrate obtained from Brazil, were investigated by untargeted metabolic profiling. Sixty-six metabolites were detected by nano-liquid chromatography coupled to a high-resolution electrospray-ionization quadrupole time-of-flight mass spectrometer (nLC-ESI-qTOF-MS) belonging to phenolic acids, coumarins, flavonoid glycosides, limonoids, terpenes, and fatty acids. Eleven metabolites were detected for the first time in *Citrus sinensis* and identified as citroside A, sinapic acid pentoside, apigenin-*C*-hexosyl-*O*-pentoside, chrysoeriol-*C*-hexoside, di-hexosyl-diosmetin, perilloside A, gingerol, ionone epoxide hydroxy-sphingenine, xanthomicrol, and coumaryl alcohol-*O*-hexoside. Some flavonoids were completely absent from the juice, while present most prominently in the *Citrus* peel, conveying more industrial and economic prospects to the latter. Multivariate data analyses clarified that the differences among orange parts overweighed the geographical source. PCA analysis of ESI-(−)-mode data revealed for hydroxylinoleic acid abundance in flavedo peel from Uruguay the most distant cluster from all others. The PCA analysis of ESI-(+)-mode data provided a clear segregation of the different *Citrus sinensis* parts primarily due to the large diversity of flavonoids and coumarins among the studied samples.

## 1. Introduction

*Citrus* trees bear some of the most popular fruits and are grown globally for food, medicinal and other industrial applications, with a total annual production of nearly 85 million tons [1]. Various species of *Citrus* genus are valuable, such as *C. medica* (citron), *C. limon* (lemon), *C. aurantium* (sour orange), *C. reticulata* (mandarin, tangerine), *C. paradisi* (grapefruit), *C. clementina* (clementine) and *C. sinensis* (sweet orange) [2]. They are either consumed as fresh fruits or after processing to juices, beverage products, and jams, with the peel being the main by-product of processing. Anatomically, the fruit consists of two parts—the outer peel and the pulp with juice sac glands [2]. The peel main parts include the outer pigmented flavedo with parenchymatous cells and cuticle, and the white albedo part lying beneath the flavedo [3]. Different plant parts find wide application in many countries in recipes to treat stomach disorders, skin inflammation, cough, muscular pain, and nausea, as well as being used as a slimming agent [4].

Phytochemical investigations have revealed the presence of bioactive coumarins (i.e., bergamottin, scopoletin, and umbelliferone), flavonoids (i.e., rutin, quercetin, and kaempferol), limonoids (i.e., limonin and nomilin), and acridone alkaloids (i.e., citruscridone and citrusinine-I) in *Citrus* plants [5]. Dietary fiber, minerals, carotenoids, and phenolic phytoconstituents, i.e., phenolic acids, flavanones, and polymethoxylated flavones, were all detected in orange peel [6]. *Citrus sinensis* var. Valencia fruits revealed superiority as an excellent source of phenolics, flavonoids and ascorbic acid compared to var. Mandora and some grapefruits grown in Cyprus [7]. Another study on var. Valencia pointed out that the flavedo part had highest vitamin C, flavones and carotenoid content, while the albedo part was the main source of flavanones and phenolics [8].

Moreover, previous clinical and animal studies have demonstrated that certain *Citrus* metabolites have antioxidant, anti-inflammatory, cytotoxic, antimicrobial, antiallergic and antiplatelet aggregation activities [9]. The metabolites in *Citrus* varieties influence their unique qualities, such as taste, appearance, and the supposed health benefits. Quantitative and qualitative differences in these metabolites are commonly affected by the cultivation environment and *Citrus* variety [10] and also vary significantly from one part to another within the *Citrus* fruit. 

Although there is a wide variety of *Citrus* fruits that were intensely investigated for their metabolic composition [11], comparative studies of metabolite profiles within different fruit parts are limited. Because metabolite variability and modification are affected by the plant’s adaptation to physiological, pathological, and diverse chemical stimuli, metabolomics enables quantitative investigation of these dynamic changes [12]. In-depth food analyses help to uncover discriminatory biomarkers, identify various pathways, find therapeutic targets, and discover new potential drug leads by evaluating and verifying the major variations in metabolite profiling [13]. 

Metabolite profiling by mass spectrometry is regarded as the most powerful analytical tool. Among the most recent detection techniques are: high-performance liquid chromatography–diode array detection (HPLC-DAD), ultra-high-performance liquid chromatography coupled to triple quadrupole mass spectrometry (UHPLC-QqQ-MS/MS), and ultra-performance liquid chromatography/quadrupole time-of-flight mass spectrometry (UPLC-QTOF-MS/MS) [14]. The analysis of the orange peel matrix has also been performed via HPLC-UV-ESI-MS/MS (high-performance liquid chromatography coupled with ultraviolet and electrospray ionization mass spectrometry) [6]. The current technique involves the use of nano-high-performance liquid chromatography (n-LC) coupled to high-resolution MS to provide fast metabolite analysis of orange peel, to the best of our knowledge, for the first time on a high sensitivity level. Previous studies have been performed via state-of-the-art nano-liquid chromatography–ESI–MS/MS technology to only separate chiral naringenin in *Citrus* pulp and peel [15]. 

By adding multivariate data analysis (MVA) techniques, a large-scale metabolomics dataset is presented in this study to provide a detailed insight into *C. sinensis* secondary metabolites. The use of MVA enables accurate specimen to specimen comparison and highlights distinctive traits [16].

The aim of this work was to investigate the differences in secondary metabolite composition in different parts of *C. sinensis* presenting the first comparative insights into metabolite profiles derived from various parts of Valencia orange fruits collected from different countries. A detailed identification of metabolites in different *C. sinensis* parts was considered together with an untargeted metabolic profile for the edible part and the peel as the main by-product during fruit processing.

## 2. Materials and Methods

### 2.1. Orange Samples and Chemicals

Valencia oranges (*C. sinensis*) from Spain and Uruguay, provided by Symrise (Holzminden, Germany) as depicted in Table 1, were cleaned and squeezed to obtain the juice, then flavedo and albedo were manually stripped by a fruit peeler. After that, all orange samples, including a Brazilian orange concentrate acquired from Symrise, were lyophilized and separately ground to fine powder by a mill rotor cyclone (Tecnal, Piracicaba, Brazil, TE-651/2). Comminuted orange samples were stored at −80 °C until subsequent analysis. All chemicals and solvents were purchased from Sigma Aldrich (Steinheim, Germany).

### 2.2. Metabolite Mass Fingerprinting

Orange specimens (2 mg), extracted by 1 mL methanol, were spiked with 4 μg mL ^−1^ hesperidin followed by sonication for 30 min, then centrifugation for 20 min at 15,000× *g* to eliminate any leftover debris. Solid-phase extraction was applied to each extract using a C_18_ cartridge (JT Baker, Phillipsburg, NJ, USA) as previously reported [17]. The resulting extracts were injected into a nano-LC system EASY-nLC II (Bruker, Bremen, Germany) equipped with a reversed phase column (150 × 0.1 mm, particle size 3 μm; Michrom Bioresources, Auburn, CA, USA) coupled to maXis impact quadrupole-time of-flight (qTOF) MS (Bruker, Bremen, Germany). A captive nano-spray ionization was operated in the negative and positive ion modes under conditions as previously reported [18]. Identification of metabolite mass fingerprints was carried out using exact parent ion masses as well as retention data, reference literature, fragmentation patterns, and the Phytochemical Dictionary of Natural Products Database (https://dnp.chemnetbase.com/ accessed on (16 March 2022)). Semi-quantification was based on the integrated peak areas of each compound after normalization to internal standard. Aiming at comparing the relative abundance of a given compound in the seven different samples, the determination of absolute concentrations by an external calibration of every compound was not required. Three independent replicates of each orange specimen were analyzed in parallel to evaluate the biological variance. 

### 2.3. Multivariate Data Analyses (MVA)

Modeling viz. principal component analysis (PCA) and orthogonal projection to latent structures-discriminant analysis (OPLS-DA) was applied to a metabolite dataset of MS abundances produced by nLC-MS either in the negative or positive ion mode via the SIMCA-P+ 13.0 software package (Umetrics, Umeå, Sweden) to pinpoint various markers characterizing each group declared with correlation (pcor) and covariance (p). The iterative permutation testing and diagnostic indices, viz. R2 and Q2 values, were used to assess the validity of models, while all variables were mean centered and Pareto scaled.

## 3. Results and Discussion

### 3.1. Metabolite Identification via nLC–ESI-MS/MS Analysis 

**Figure 1:**
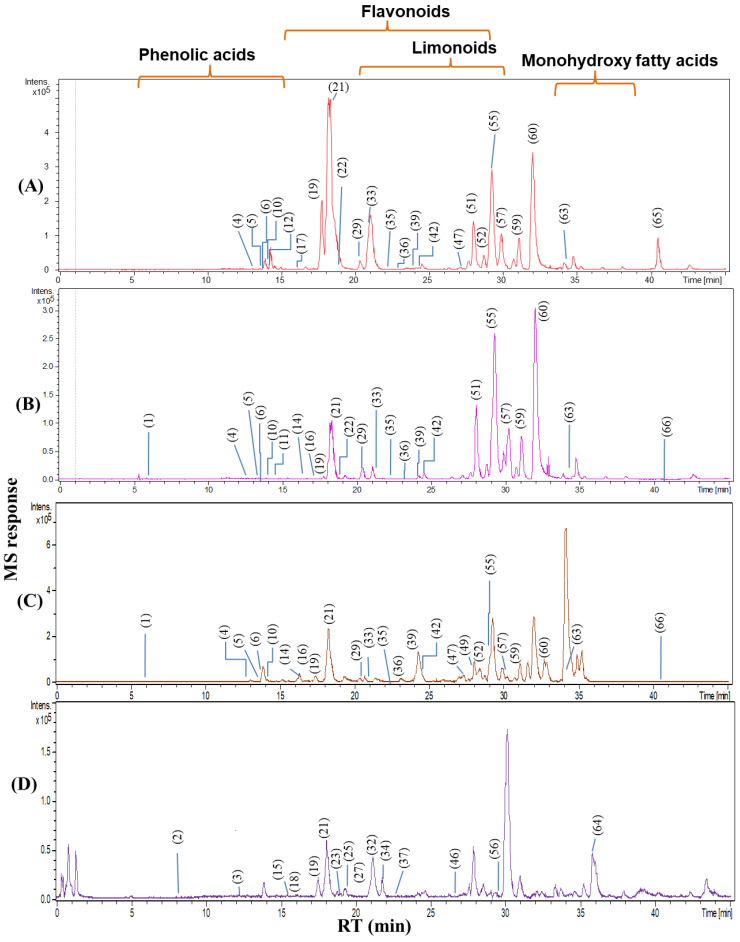
Representative nLC-MS base peak chromatogram of orange parts extracted by methanol on negative ionization (**A**) albedo from Uruguay, (**B**) orange concentrate from Brazil, (**C**) flavedo from Uruguay and on positive ionization, and (**D**) juice from Uruguay. For peak numbers, refer to Table 2.

nLC-ESI-MS/MS analyses (Figure 1) of *Citrus* samples resulted in the identification of 66 metabolites, categorized into seven classes: phenolic acids, terpenes, limonoids, coumarins, flavonoids, fatty acids and nitrogenous compounds. The elution order of metabolites followed a sequence of decreasing polarity, whereby phenolic acids eluted first, followed by coumarins, flavonoid glycosides, limonoids, free aglyca and fatty acids. Samples were analyzed in both the negative and positive ionization modes to provide a greater coverage of the metabolome. Fatty acids and flavonoids were preferentially ionized under negative ionization conditions, while coumarins, limonoids and nitrogenous compounds showed better ionization in the positive mode. The list of identified compounds along with their retention time, characteristic molecular and fragment ions and occurrence is presented in Table 2.

#### 3.1.1. Identification of Organic Acids and Phenolics

A number of organic acids were eluted in the first part of the nLC-ESI-MS/MS-chromatogram, as revealed from their MS spectral data. In the analyzed *Citrus* samples, the most representative phenolic and organic acids were citric, ferulic, sinapic acids and their glycosides. Peak 1—for peak numbers refer to Table 2—[(M−H)^−^ *m/z* 191.0189 (C_6_H_7_O_7_)^−^] was identified as citric acid previously reported in *Citrus limon* ethanolic extract [19]. Peaks **6** and **8** (Suppl. Figure S1), [(M−H)^−^ *m/z* 355.103 (C_16_H_19_O_9_)^−^] were annotated as ferulic acid hexoside and sinapic acid pentoside, respectively. Having the same molecular formula; peak **6** yielded a main fragment ion at *m/z* 193 [M−H−162 (hexose)]^−^relative to ferulic acid, while peak **8** showed a fragment ion at *m/z* 223 [M−H−132 (pentose)]^−^ relative to sinapic acid. Sinapic acid pentoside was detected for the first time in all of the examined *Citrus* samples except *Citrus sinensis* juice obtained from Uruguay (JU). In addition, citric acid was absent in the albedo of both suppliers (AS: albedo from Spain, and AU: albedo from Uruguay), while sinapoyl hexoside was absent in the juice (JS: juice from Spain, and JU: juice from Uruguay). Peak **17** [(M−H)^−^ *m/z* 311.1139 (C_15_H_19_O_7_)^−^] with product ion at *m/z* 161 corresponding to negatively ionized hexose, was annotated as coumaryl alcohol-*O*-hexoside, first time to be reported in all *Citrus* samples. Peak **55** [(M−H)^−^ *m/z* 293.1763 (C_17_H_25_O_4_)^−^] was a major constituent detected in all analyzed samples and identified as gingerol, a pungent principle previously detected in the peel of *Citrus reticulata* [20], but detected for the first time in *Citrus sinensis* and most prominent in albedo from Uruguay and juice from Spain (AU and JS). Ferulic and *p*-coumaric acid were major hydroxycinnamic acids detected in both free and bound form in the methanolic extracts of bitter orange (*Citrus microcarpa*) peel [6].

#### 3.1.2. Identification of Coumarins

Peak **11** [(M−H)^−^ *m/z* 205.0504 (C_11_H_9_O_4_)^−^] yielded fragment ions at *m/z* 175 [M−H−30 (C_2_H_6_)]^−^ and *m/z* 101 corresponding to the loss of CO_2_ from the pyrone ring, and was identified as citropten, the most abundant coumarin in *Citrus* (Suppl. Figure S2) [19]. Peak **9** [(M−H)^−^ *m/z* 175.0392 (C_10_H_7_O_3_)^−^] was identified as methoxycoumarin and detected in all *Citrus* samples. Peak **26** [(M−H)^−^ *m/z* 367.0668 (C_16_H_15_O_10_)^−^ showed a characteristic fragment ion at *m/z* 191 [M−H−176 (glucuronyl)]^−^ was identified as scopoletin-*O*-glucuronide. The flavedo part from Uruguay showed the highest level of the identified coumarins. This finding was in accordance with results previously reported by Liu et al. (2017) showing that the highest coumarin content was found in the flavedo part among different studied *Citrus* cultivars [21], conveying a particular interest regarding the presumed anticancer and antidiabetic effects of this part.

**Table 2:** Metabolites annotated in various orange samples via nLC -ESI-MS/MS using both the positive and negative ionization modes.

No.	Rt (min)	Compound Name	Chemical Class	[M−H]^−^/ [M + H]^+^	Molecular Formula	Mass Error	MS/MS frag.	Ref.	AS	AU	CB	FS	FU	JS	JU
1.	5.7	Citric acid	Organic acid	191.0189	C_6_H_7_O_7_^−^	4.3	111, 87	[19]	-	-	++	+	+	++	++
2.	11.1	Feruloylagmatine	Cinnamic acid amide	307.177	C_15_H_23_N_4_O_3_^+^	1.7	264, 177		++	++	+	+	-	+	+
3.	13.0	Hydroxy-phenyl-valeric acid-*O*-sulphate	Sulphate ester	275.0607	C_11_H_15_O_6_S^+^	8.4	195		+	-	+	+	++	+	+
4.	13.2	Dihydroxymegastigmadienone -*O*-hexoside (Citroside A)	Terpene	385.1860	C_19_H_29_O_8_^−^	0.8	223, 153	[22]	++	+	+	+	++	-	-
5.	13.9	Sinapoyl hexoside	Phenolic acid	385.1141	C_17_H_21_O_10_^−^	−1.8	223, 190	[19]	++	+	+	+	++	-	-
6.	13.9	Ferulic acid hexoside	Phenolic acid	355.1030	C_16_H_19_O_9_^−^	0.0	193, 175, 160	[19]	++	++	++	++	++	+	++
7.	13.7	Citropten	Coumarin	205.0504	C_11_H_9_O_4_^−^	1.1	175, 101	[19]	+	+	+	+	++	+	+
8.	13.9	Sinapic acid pentoside	Phenolic acid	355.1037	C_16_H_19_O_10_^−^	−0.7	223, 205	[23]	++	+	+	++	+	+	-
9.	13.9	Methoxycoumarin	Coumarin	175.0392	C_10_H_7_O_3_^−^	0.2	-	[24]	+	+	+	+	+	+	+
10.	14.0	Sinapic acid	Phenolic acid	223.0607	C_11_H_11_O_5_^−^	0.2	-	[19]	++	++	++	+	++	+	+
11.	14.2	Di-hexosyl-diosmetin	Flavone	625.1757	C_28_H_33_O_16_^+^	−1	-	[25]	+	+	+	++	++	-	-
12.	14.3	Naringenin-*O*-hexoside	Flavanone	433.1145	C_21_H_21_O_10_^−^	1.2	271	[26]	++	++	-	+	-	-	-
13.	14.7	Limononic acid	Limonoid degradation	183.1024	C_10_H_15_O_3_^−^	1.6	-		++	+	+	+	++	-	+
14.	13.2	Methyl epijasmonate	Fatty acid degradation	223.1337	C_13_H_19_O_3_^−^	1.3	-	[27]	++	++	+	+	++	+	+
15.	16.4	Apigenin-di-*C*-hexoside	Flavone	595.1654	C_27_H_31_O_15_^+^	1.5	385	[26]	+	+	+	++	++	+	+
16.	16.5	Apigenin-di-*O*-hexoside	Flavone	593.1502	C_27_H_29_O_15_^−^	8.6	431, 269	[26]	+	-	+	+	++	-	-
17.	16.6	Coumaryl alcohol-*O*-hexoside	Cinnamyl alcohol glycoside	311.1139	C_15_H_19_O_7_^−^	1.1	269, 161		+	+	+	+	++	+	+
18.	16.8	Apigenin-*C*-hexosyl-*O*-pentoside	Flavone	563.1408 565.1550	C_26_H_27_O_14_^−^ C_26_H_29_O_14_^+^	0.3 0.3	311	[28]	-	-	+	+	++	+	+
19.	17.5	Naringenin-*O*-hexosyldeoxyhexoside	Flavanone	579.1720 581.1867	C_27_H_31_O_14_^−^ C_27_H_33_O_14_^+^	1.0 −0.5	271 419, 273	[29]	++	++	++	+	+	+	+
20.	17.6	Chrysoeriol-*C*-hexoside	Flavone	463.1238	C_22_H_23_O_11_^+^	−0.1	343	[26]	+	+	+	++	++	+	+
21.	18.2	Hesperidin (Hesperetin-*O*-hexosyldeoxyhexoside)	Flavanone	609.1833 611.1971	C_28_H_33_O_15_^−^ C_28_H_35_O_15_^+^	−2.2 0.1	301 -	[29]	++	++	++	++	++	++	+
22.	18.3	Limonin-hexoside	Limonoid	649.2507	C_32_H_41_O_14_^−^	0.7	-	[30]	+	+	+	+	+	+	+
23.	18.7	Hesperetin-*O*-deoxyhexoside	Flavanone	449.1625	C_22_H_25_O_10_^+^	−0.4	303	[19]	++	+	-	+	+	-	-
24.	19.1	Azelaic acid	Dicarboxylic acid	187.0969	C_9_H_15_O_4_^−^	2.1	169, 125		+	+	+	+	+	+	+
25.	19.3	Deacetylnomilin	Limonoid	473.2167	C_26_H_33_O_8_^+^	−1.7	455, 411, 161		++	+	+	+	+	+	+
26.	19.4	Scopoletin-*O*-glucuronide	Coumarin	367.0668	C_16_H_15_O_10_^−^	−0.1	191		+	+	+	+	++	+	+
27.	20.2	Pyridoxamine phosphate	Vitamin B6 phosphate	249.0614	C_8_H_14_N_2_O_5_P^+^	−8.4	169, 81	[31]	+	+	++	+	+	+	+
28.	20.3	Unknown	Alkaloid	368.1921	C_15_H_30_NO_9_^+^	−13	-		+	+	-	++	++	+	-
29.	20.4	Decatrienoic acid	Fatty acid	165.0913	C_10_H_13_O_2_^−^	−5.0	-		+	+	+	+	+	+	+
30.	21.0	Sakuranetin-*O*-hexosyl-*O*-deoxyhexoside	Flavanone	595.2020	C_28_H_35_O_14_^+^	−1.2	433, 287	[28]	++	++	-	+	+	-	-
31.	20.7	Hydroxyhexadecanedioic acid	Fatty acid	303.2171	C_16_H_31_O_5_^−^	1.1	259, 214	[32]	+	-	-	+	++	-	-
32.	20.9	Sakuranetin-*O*-hexosyl deoxyhexoside	Flavanone	593.1835 595.2018	C_28_H_33_O_14_^−^ C_28_H_35_O_14_^+^	1.7 −1.5	285		+	+	++	++	++	++	+
33.	21.1	Nomilin-hexoside	Limonoid	693.2723	C_34_H_45_O_15_^−^	5.9	159	[19]	++	++	+	+	+	++	+
34.	21.2	Sakuranetin	Flavanone	285.0770 287.1042	C_16_H_13_O_5_^−^ C_16_H_15_O_5_^+^	2.5 0.7	255 237	[29]	++	++	+	+	+	+	+
35.	22.5	Perilloside A	Terpene	313.1651	C_16_H_25_O_6_^−^	−0.3	-	[33]	++	+	+	++	+	+	-
36.	23.0	Trihydroxy-octadecadienoic acid (trihydroxy-linoleic acid)	Fatty acid	327.2175	C_18_H_31_O_5_^−^	0.9	-	[28]	+	+	+	+	++	+	+
37.	23.7	Hydroxy-sphingenine	Ceramide	316.2827	C_18_H_38_NO_3_^+^	−6.1	-	[34]	+	+	++	+	+	+	-
38.	23.9	Linoleamide	Fatty acid amide	280.2677	C_18_H_34_NO^+^	1.4	-	[35]	+	-	-	+	-	++	-
39.	24.1	Trihydroxy-octadecenoic acid	Fatty acid	329.2333	C_18_H_33_O_5_^−^	1.5	-		+	+	+	+	++	+	+
40.	24.2	Demethylnobiletin	Methoxy-flavone	389.1234	C_20_H_21_O_8_^+^	−0.8	-	[36]	-	-	-	+	++	-	-
41.	24.3	Methyl dihydrojasmonate	Organic acid	225.1496	C_13_H_21_O_3_^−^	−0.4	-		+	+	+	+	+	+	+
42.	17.7	Naringenin	Flavanone	271.0606	C_15_H_11_O_5_^−^	0.0	-	[37]	+	+	++	++	+	++	++
43.	26.1	Nobiletin	Methoxy-flavone	403.1385	C_21_H_23_O_8_^+^	−1.2	388, 373, 342	[38]	+	+	+	++	++	-	-
44.	18.5	Hesperetin	Flavanone	301.0717	C_16_H_13_O_6_^−^	0.9	-	[29]	++	++	+	+	+	+	+
45.	26.5	Unknown	Methoxy-flavone	403.1568	C_25_H_23_O_5_^+^	7.4	373		-	-	+	+	++	+	+
46.	27.2	Tangeretin	Methoxy-flavone	373.1312	C_20_H_21_O_7_^+^	6.7	343, 312	[39]	+	+	+	+	+++	+	+
47.	27.7	Methyl-dodecadienoate	Fatty acid ester	209.1548	C_13_H_21_O_2_^−^	2.4	-		+	+	+	+	+	++	+
48.	27.8	Unknown	Alkaloid	272.1862	C_14_H_26_NO_4_^+^	−1.8	255, 237		++	+	+	+	+	+	+
49.	27.9	Dimethylkaempferol	Methoxy-flavone	313.0718	C_17_H_13_O_6_^−^	1.9	163, 117	[40]	-	+	+	++	++	-	-
50.	28.0	γ-Lactone hydroxy-dodecenedioic acid methyl ester	Fatty acid ester	253.1443	C_18_H_15_O_7_^−^	0.0	209		+	+	+	+	+	+	+
51.	28.1	Limonin	Limonoid	469.1869 471.2018	C_26_H_29_O_8_^−^ C_26_H_31_O_8_^+^	1.5 0.2	- 453, 161	[30]	++	+	+	+	+	+	+
52.	28.3	Methyllimonexic acid	Limonoid	515.1925	C_27_H_31_O_10_^−^	1.6	469, 229	[41]	++	+	-	+	+	+	+
53.	28.4	Dihydroxytrimethoxyflavone (xanthomicrol)	Methoxy-flavone	343.0826	C_18_H_15_O_7_^−^	0.2	298, 270, 242		+	-	+	++	++	-	-
54.	29.0	Tetramethoxyflavone (Tetra-*O*-methylscutellarein)	Methoxy-flavone	343.1201	C_19_H_19_O_6_^+^	7.2	313, 282		+	+	+	++	++	-	-
55.	29.2	Gingerol	Phenylpropen-oid polyketide	293.1763	C_17_H_25_O_4_^−^	3.8	193	[20]	+	++	+	+	+	++	+
56.	29.5	Heptamethoxyflavone	Methoxy-flavone	455.1313 433.1511	C_22_H_24_O_9_Na^+^ C_22_H_25_O_9_^+^	−1.1 −4.1	433 403	[42]	-	-	+	+++	++	-	-
57.	29.9	Ionone epoxide	Terpene	207.1392	C_13_H_19_O_2_^−^	−3.4	-	[43]	+	+	+	+	+	+	+
58.	28.7	Hydroxy-tetradecatrienoyl glycerol	Acylglycerol	311.1857	C_17_H_27_O_5_^−^	−2.2	-		+	+	+	+	++	+	+
59.	31.6	Dihydroxyoctadecadienoic acid	Fatty acid	311.2225	C_18_H_31_O_4_^−^	1.2	211, 171, 129		+	+	+	++	++	+	+
60.	32.0	*N*-Phenylacetylglycine	Nitrogenous compound	194.0821	C_10_H_12_NO_3_^−^	0.8	-		+	+	++	+	+	++	+
61.	32.5	Hydroxy-oxohexadecanoic acid	Fatty acid	285.2069	C_16_H_29_O_4_^−^	0.7	-	[44]	++	++	+	+	+	+	+
62.	32.5	Hydroxylinolenic acid	Fatty acid	293.2115	C_18_H_29_O_3_^−^	−2.4	275, 235	[45]	+	+	-	+	++	-	-
63.	34.1	Hydroxyoctadecadienoic acid (Hydroxylinoleic acid)	Fatty acid	295.2276	C_18_H_31_O_3_^−^	1.6	-		+	+	+	+	++	+	+
64.	35.7	Heptyl caffeate	Cinnamic acid ester	301.1421 279.1600	C_16_H_22_O_4_Na^+^ C_16_H_23_O_4_^+^	1.7 −3.3	279		+	+	-	++	++	+	++
65.	39.1	Erucamide (Docosenamide)	Fatty acid amide	338.3428	C_22_H_44_NO^+^	−3.1	321	[35]	-	-	+	+	-	++	-
66.	40.5	Linoleic acid	Fatty acid	279.2326	C_18_H_31_O_2_^−^	1.3	-	[45]	+	++	+	+	+	+	+

AS: Albedo from Spain; AU: Albedo from Uruguay; CB: concentrate from Brazil; FS: Flavedo from Spain; FU: Flavedo from Uruguay; JS: Juice from Spain; JU: Juice from Uruguay. Signs (-, +, ++ and +++) indicate relative semi-quantification of each compound based on the integrated peak areas of the total ion; - = absent, + = present, ++ = present in higher amount, and +++ = present in highest amount.

#### 3.1.3. Identification of Flavonoids

A total of 22 flavonoid derivatives were identified with better ionization in the negative ion mode as a result of their rapid deprotonation ability. Peak **20** [(M + H)^+^ *m/z* 463.1238 (C_22_H_23_O_11_)^+^], with a fragment ion at *m/z* 343 [M + H−120]^+^ was identified as chrysoeriol-*C*-hexoside. Peak **15** [(M + H)^+^ *m/z* 595.1654 (C_27_H_31_O_5_)^+^] showed a fragment ion at *m/z* 385 [M + H−120−90]^+^, indicating di-*C*-hexoside, and was identified as apigenin-di-*C*-hexoside (vicenin-2). Vicenin-2 was previously identified in the peels and edible pulp of *C. aurantiifolia,* and *C. unshiu* [46]. Both flavones, chrysoeriol-*C*-hexoside and vicenin-2, were detected in all the analyzed samples with relatively high concentration in flavedo peel of both suppliers.

Peaks **12** and **19** showed the same deprotonated aglycone fragment ion at *m/z* 271, and were identified as naringenin derivatives. Peak **12** [(M−H)^−^ *m/z* 433.1145 (C_21_H_21_O_10_)^−^] with MS^2^ fragment at *m/z* 271 [M−H−162 (hexose)]^−^ was identified as naringenin-*O*-hexoside. Peak **19** [(M + H)^+^ *m/z* 581.1867 (C_27_H_33_O_14_)^+^] showed fragment ions (Suppl. Figure S3) at *m/z* 419 [M + H−162 (hexose)]^+^ followed by successive loss of a deoxyhexose at *m/z* 273 [M + H−162 (hexose)−146 (deoxyhexose)]^+^, while peak **31** showed a base signal at *m/z* 273.0756 [M + H−308 (rutinose)]^+^ indicating that both carbohydrate moieties were linked through an -*O*-glycosidic bond [47]. Thus, peak **19** was identified as naringenin-*O*-hexosyldeoxyhexoside.

Likewise, Peaks **16** and **18** showed the same deprotonated fragment ion at *m/z* 269 indicating apigenin derivatives. Peak **16** [(M−H)^−^ *m/z* 593.1502 (C_27_H_29_O_15_)^−^] with MS^2^ fragments at *m/z* 431 [M−H−162 (hexose)]^−^ and *m/z* 269 [M−H−162 (hexose)−162 (hexose)]^−^, indicating successive losses of two hexose moieties, was identified as apigenin-di-*O*-hexoside previously detected in different *Citrus* species [26]. Peak **18** [(M−H)^−^ *m/z* 563.1408 (C_26_H_27_O_14_)^−^]/[(M + H)^+^ *m/z* 565.1550 (C_26_H_29_O_14_)^+^] was a mixed *O-C* flavone, with characteristic fragment ion at *m/z* 311 [M−H−120 (cross ring cleavage of *C*-hexosyl)−132 (pentose)]^−^. It was identified as apigenin-*C*-hexosyl-*O*-pentoside, first reported in *Citrus sinensis* (Suppl. Figure S4). In the studied samples, flavedo peel from Uruguay was found more enriched in apigenin derivatives, while naringenin derivatives were found most abundant in the albedo part of both suppliers.

Peaks **30** showed deprotonated fragment ion at *m/z* 285, indicating sakuranetin derivatives. Peak **30** showed fragment ions at *m/z* 433 [M + H−162 (hexose)]^+^ followed by successive loss of a deoxyhexose at *m/z* 287 [M + H−162 (hexose)−146 (deoxyhexose)]^+^. Thus, peak **30** was identified as sakuranetin-*O*-hexosyl-*O*-deoxyhexoside.

Peak **21** [(M−H)^−^ *m/z* 609.1833 (C_28_H_33_O_15_)^−^]/[(M + H)^+^ *m/z* 611.1971 (C_28_H_35_O_15_)^+^] showed a MS^2^ fragment ion in the negative ionization mode at *m/z* 301 [M−H−308 (rutinose)]^−^ and was the main flavanone glycoside identified in all samples (Suppl. Figure S5). It was annotated as hesperidin, previously reported as the main flavanone in the peel of *Citrus sinensis* L. varieties, which suffers dramatic losses in filtered peel juice due to its relatively low water solubility [48]. Hesperidin is well known for its supposed effects on health including antimicrobial, anticancer, antihypertensive and antiulcer effects, thus attracting medicinal interest to orange peel [49].

A number of polymethoxy flavones (PMF) were identified in the studied samples being more readily ionized in the positive ion mode than in the negative mode. In general, the PMF MS^2^ spectra had characteristic fragments at [M + H−nCH_3_]^+^, [M + H−2CH_3_−CO]^+^ and [M + H−2CH_3_−H_2_O]^+^ [50]. Peak **43** [(M + H)^+^ *m/z* 403.1385 (C_21_H_23_O_8_)^+^], peak **46** [(M + H)^+^ *m/z* 373.1312 (C_20_H_21_O_7_)^+^] and peak **53** [(M−H)^−^ *m/z* 343.0826 (C_18_H_15_O_7_)^−^] showed typical fragmentation patterns for PMF and were identified as nobiletin, tangeretin and dihydroxytrimethoxy-flavone, respectively. Xanthomicrol (dihydroxytrimethoxy-flavone) was previously isolated from *Citrus sudachi* [51] but reported herein for first time in *C. sinensis*. Likewise, peak **54** [(M + H)^+^ *m/z* 343.1201 (C_19_H_19_O_6_)^+^] and peak **56** [(M + H)^+^ *m/z* 433.1511 (C_22_H_25_O_9_)^+^] were annotated as tetra-*O*-methylscutellarein and heptamethoxyflavone, respectively. In addition to xanthomicrol, other hydroxylated PMFs were detected as demethylnobiletin (peak **40**), and dimethylkaempferol (peak **49**). Generally, PMFs exhibited extensive range of biological actions, i.e., antiatherogenic, and anti-inflammation activities [52]. However, hydroxylated PMFs revealed potent antineoplastic effects against various cancer types [53]. Flavedo peel from Uruguay was the richest part in methoxy flavones in contrast to the juice from both suppliers. The flavedo extracts of various *Citrus* fruits encompassed flavanone glycosides (poncirin, hesperidin, neohesperidin, dydimin, naringin, narirutin, and neoeriocitrin), flavonol glycosides (rutin), and flavone glycosides (diosmin, rhoifolin, and isorhoifolin) [6].

#### 3.1.4. Identification of Limonoids and Terpenes

Limonoids are tetranortriterpenoids, found extensively in Rutaceae and Meliaceae [54]. They are widely distributed in different *Citrus* fruits, such as grapefruit (*Citrus paradisi*), sweet orange (*Citrus sinensis*), sour orange (*Citrus aurantium*), lemon (*Citrus limon*) and lime (*Citrus aurantiifolia*) [55]. Water-insoluble limonoid aglycones are mainly distributed within seeds and peels. In contrast, the water-soluble limonoid glycosides are more abundant within juices and pulps [56]. In the studied *Citrus* samples, limonoids occur in significant amounts as aglycone and glycoside forms. Peak **25** [(M + H)^+^ *m/z* 473.2167 (C_26_H_33_O_8_)^+^] showed MS^2^ fragments at *m/z* 455 [M + H−18 (H_2_O)]^+^, 411 [M + H−62 (CH_2_O_3_)]^+^ and the characteristic ion for *Citrus* limonoids at *m/z* 161 attributed to the furan ring bound to a lactone ring moiety [57] and was identified as deacetylnomilin (Suppl. Figure S6). It was found more abundant in the peel than in juice or concentrate; this may be related to its relatively low water solubility. Peak **51** with protonated and deprotonated molecular ions [(M + H)^+^ *m/z* 471.2018 (C_26_H_31_O_8_)^+^]/[(M−H) *m/z* 469.1869 (C_26_H_29_O_8_)^−^] showed a fragment ion *m/z* 453 [M + H−18 (H_2_O)]^+^ and the characteristic fragment ion for *Citrus* limonoids at *m/z* 161. It was identified as limonin, a well-known limonoid that possesses various biological, mainly anti-inflammatory activities [58]. Limonin was detected in more abundant content in albedo from Spain (AS), suggestive to have a slightly bitter taste compared to albedo from Uruguay (AU) upon providing a likely sustainable source of food additive [59].

Terpenes are important for plant aroma and flavor playing key roles in fruit quality, plant defense and pollinator attraction. A total of three terpenes was detected ionized most preferentially in the negative ionization mode, from which a gluco-conjugated megastigmadienone was identified as peak **4** [(M−H)^−^ *m/z* 385.1860 (C_19_H_29_O_8_)^−^] known as citroside A. Its MS^2^ fragments showed the characteristic fragment ion at *m/z* 223 [M−H−162 (hexose)]^−^ relative to the loss of a hexose moiety. Citroside A was reported previously to be a precursor of damascenone and 3-hydroxydamascone, two important industrial flavor compounds. It has been reported in *Citrus unshiu* leaves, *Solanum quitoense* leaves [60] and *Gynostemma pentaphyllum* [61]. Herein it was detected for the first time in the peel of *Citrus sinensis* being more prominent in albedo peel from Spain and flavedo peel from Uruguay (AS and FU). A number of monoterpenes belonging to different classes were identified: Peak **35** [(M−H) *m/z* 313.1651 (C_16_H_25_O_6_)^−^] was a monoterpene glycoside identified as perilloside A, detected for the first time in *Citrus* and found most prominent in albedo and flavedo peel from Spain (AS and FS), suggestive to be a marker to distinguish between peels from Spain and Uruguay. Peak **57** [(M−H)^−^ *m/z* 207.1392 (C_13_H_19_O_2_)^−^] was an apo-carotenoid monoterpene identified as ionone epoxide, a flavoring substance with a fruity and woody flavor previously identified in many foods, such as apricot, raspberry, tea and lemon balm [43].

#### 3.1.5. Identification of Fatty Acids and Fatty Acid Amides

Saturated and unsaturated fatty acids in addition to low molecular mass amide compounds were detected in several orange peel compartments. For details, refer to Supplementary Materials [28,35,44].

#### 3.1.6. Identification of Nitrogenous Compounds

Other metabolites containing nitrogen were detected at trace levels. For details, refer to Supplementary Materials [34].

### 3.2. Multivariate Data Analyses of Orange Samples

The datasets encompassed a total of 42 nLC-ESI-MS/MS runs (seven different orange samples with three biological replicates each in both the negative and positive ionization modes). Thus, unsupervised and supervised multivariate data analyses were adopted to simplify interpretation of such complex datasets allowing better biomarkers characterization and sample classification [62]. Principal component analysis (PCA), as an unsupervised approach, was reported to evaluate the variance within various samples involving no prior knowledge of the datasets [63]. Table 1 shows the color-coded source of the *Citrus sinensis* specimens compared.

**Figure 2:**
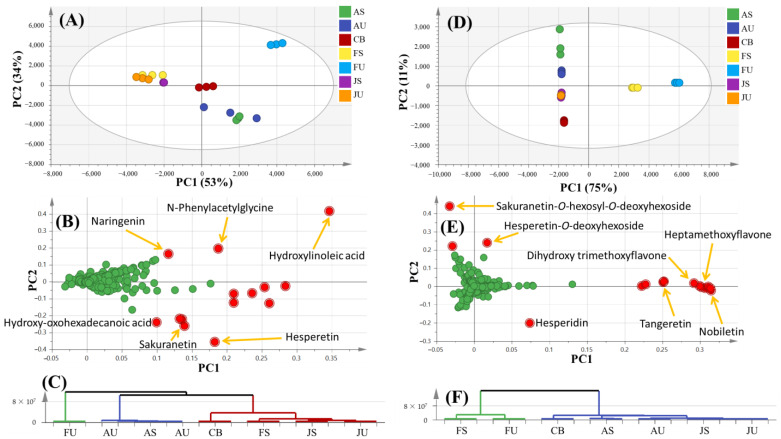
MS-based PCA of all orange samples (*n* = 3) negative ionization (**A**) score plot, (**B**) relevant loading plot and (**C**) HCA; positive ionization (**D**) score plot, (**E**) relevant loading plot and (**F**) HCA. AS: Albedo from Spain; AU: Albedo from Uruguay; CB: concentrate from Brazil; FS: Flavedo from Spain; FU: Flavedo from Uruguay; JS: Juice from Spain; JU: Juice from Uruguay.

PCA on negative ionization runs as depicted in Figure 2A–C for all orange samples was illustrated by PC1 and PC2 accounting for 87% of the total variance (Figure 2A). Along PC1, there was a clear separation between flavedo specimens from Spain (FS) and Uruguay (FU), while the latter was distinguished from the other samples and located at upper right quadrant with positive score values, suggesting the impact of geographical source based on their metabolites. The albedo samples were positioned at the lower right quadrant, whereas juice samples were located at upper left quadrant with negative PC1 values. Conversely, orange concentrate from Brazil (CB) appeared near the origin (Figure 2A). The respective loading plot demonstrated that mono-methoxy flavonoids, i.e., hesperetin and sakuranetin along with hydroxy-oxohexadecanoic acid were found more abundant in albedo specimens (Figure 2B). On the other hand, hydroxylinoleic acid was major contributor to flavedo from Uruguay (FU) being the most distant data point. Naringenin was responsible for the clustering of orange juice of both suppliers and flavedo from Spain (FS) in the upper left quadrant, while *N*-phenylacetylglycine was more abundant in juice from Spain (JS) and orange concentrate (CB). The dendrogram of HCA (Hierarchical clustering analysis), as depicted in Figure 2C, revealed that flavedo from Uruguay (FU) represented one cluster, whereas the other cluster was divided into albedo specimens at one sub-group and the other sub-group encompassed the remaining samples.

Likewise, PCA on positive ionization as illustrated in Figure 2D–F revealed 86% of the total variance, albeit with better segregation (Figure 2D) compared to its negative ionization counterpart. Particularly, all replicates from each orange sample were tightly clustered indicating the superb reproducibility of the experimental analysis in both the negative and positive ionization modes. In agreement with the previous PCA model, flavedo from Uruguay (FU) was also the most distant group. Flavedo specimens showed positive score values separable from other orange parts, while orange juice and concentrate had negative score values. Albedo from both countries (AU and AS) was separated in the upper left quadrant. The respective loading plot (Figure 2E) demonstrated high levels of nobiletin, tangeretin, dihydroxytrimethoxyflavone and heptamethoxyflavone in flavedo samples. Albedo specimens were enriched in sakuranetin-*O*-hexosyl-*O*-deoxyhexoside and hesperetin-*O*-deoxyhexoside, whereas high levels of hesperidin were observed in orange juice and concentrate. The HCA dendrogram, as depicted in Figure 2F, revealed that the flavedo from both suppliers represented one cluster, while the other cluster included the remaining orange parts.

**Figure 3:**
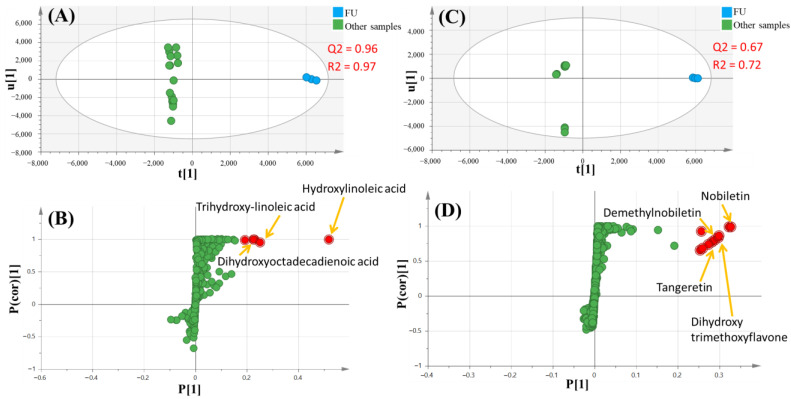
MS-based OPLS of flavedo from Uruguay (FU) against all other samples negative ionization (**A**) score plot and (**B**) relevant loading S-plot; positive ionization (**C**) score plot and (**D**) relevant loading S-plot.

OPLS-DA (orthogonal projection to latent structures-discriminant analysis) as a supervised approach was reported to possess a great potentiality maximizing the segregation of overlapping sample groups by identifying chemical determinants [64]. Being the most distant cluster, flavedo from Uruguay (FU) was further subjected to OPLS-DA against all other specimens (Figure 3) to assess sample discrimination with *p* value less than 0.001. The first OPLS model on negative ionization (Figure 3A) exhibited 0.96 model predictability (Q2) and 97% total variance (R2). The relevant loading S-plot showed that FU included abundant levels of hydroxylated fatty acids, i.e., hydroxylinoleic, trihydroxy-linoleic, and dihydroxyoctadecadienoic acids (Figure 3B). Conversely, the second OPLS model on positive ionization (Figure 3C) exhibited 0.62 model predictability (Q2) and 72% total variance (R2). The relevant loading S-plot revealed that nobiletin, dihydroxy-trimethoxyflavone, demethylnobiletin and tangeretin were detected in higher levels in FU (flavedo from Uruguay) (Figure 3D). Then, several OPLS models were constructed (Suppl. Figures S7–S10) with *p* value less than 0.001 to assess chemical determinants in orange parts derived from the various countries. Hence, a model of flavedo from Uruguay (FU) against its counterpart from Spain (FS) was performed (Suppl. Figure S7). On negative mode, OPLS model (Suppl. Figure S7A) revealed the enrichment of flavedo from Uruguay (FU) in hydroxyl-linoleic acid, dimethylkaempferol, apigenin-di-*O*-hexoside and citropten (Suppl. Figure S7B). Notably, hydroxyl fatty acids other than hydroxylinoleic acid did not appear in this model, which was attributed to their similar levels present in both samples. Conversely, OPLS model in positive mode (Suppl. Figure S7C) exhibited the enrichment of flavedo from Uruguay (FU) in nobiletin, demethylnobiletin, and tangeretin, whereas heptamethoxyflavone was abundant in FS (Suppl. Figure S7D). Another OPLS model was performed with albedo from Uruguay (AU) versus its counterpart from Spain (AS) (Suppl. Figure S8). OPLS model in negative ionization (Suppl. Figure S8A) demonstrated the particular abundance of naringenin-*O*-hexoside and linoleic acid in albedo from Uruguay (AU), while hesperetin, sakuranetin and hydroxy-oxohexadecanoic acid were found more abundant in albedo from Spain (Suppl. Figure S8B). In positive ionization, the OPLS model (Suppl. Figure S8C) showed significantly higher levels of glycosyl flavonoids, i.e., sakuranetin-*O*-hexosyl-*O*-deoxyhexoside, limonin and hesperetin-*O*-deoxyhexoside in AS (albedo from Spain) (Suppl. Figure S8D). Further and for better discrimination assessment of orange juices derived from various sources, the OPLS model in negative mode (Suppl. Figure S9A) revealed abundant levels of *N*-phenylacetylglycine, nomilin-hexoside and gingerol in orange juice from Spain (JS) (Suppl. Figure S9B). Lastly, the OPLS model including orange juice from both suppliers and orange concentrate from Brazil (CB) was implemented in positive mode (Suppl. Figure S10A). The relevant loading plot (Suppl. Figure S10B) showed that hesperidin, hydroxy-sphingenine and pyridoxamine phosphate amounted for the major metabolites in orange concentrate (CB). The obvious segregation between orange juice from Uruguay (JU) and Spain (JS) was ascribed to the abundance of heptyl caffeate in the former, while the later was enriched in nomilinhexoside and sakuranetin-*O*-hexosyl-deoxyhexoside.

## 4. Conclusions

In this study, the metabolites in the orange peels (albedo and flavedo parts) together with the juice and concentrate from different suppliers were systematically analyzed and identified using state-of-the-art nLC-ESI-MS/MS-based, widely non-targeted metabolome analysis. A total of 66 metabolites were annotated, 37 of which were compounds shared by all samples. Furthermore, 29 differential metabolites were detected, 15 of which were mainly flavonoids and completely absent from the juice. A total of eleven metabolites were detected for the first time in *Citrus sinensis:* citroside A, sinapic acid pentoside, di-hexosyl-diosmetin, apigenin-*C*-hexosyl-*O*-pentoside, chrysoeriol-*C*-hexoside, perilloside A, hydroxy-sphingenine, xanthomicrol, coumaryl alcohol-*O*-hexoside, gingerol and ionone epoxide. The annotation of the novel metabolites warrants in-depth functional genomic mining to identify their various biosynthetic pathways. Comparing different fruit parts, a number of flavonoids with proposed preventive therapies against some diseases, i.e., naringenin-*O*-hexoside, hesperetin-*O*-deoxyhexoside, sakuranetin-*O*-hexosyl-*O*-deoxyhexoside, and demethylnobiletin, were completely absent from the juices, but present most prominent in the peel. *Citrus* peel is thus considered a renewable bio-resource of functional foods. In addition, hesperetin-*O*-deoxyhexoside, sakuranetin-*O*-hexosyl-*O*-deoxyhexoside and hydroxylinolenic acid were detected only in albedo and flavedo of both suppliers, albeit absent in orange juices and concentrate, suggestive to be a distinctive marker for orange peel and to increase the potential transform of *Citrus* by-products into valuable food ingredients, nutraceuticals, and perhaps even pharmaceuticals.

The comprehensive nLC-ESI-MS/MS metabolic profiling followed by multivariate data analyses suggested that the differences between orange parts were much more obvious than the geographical source. Generally, albedo was richer in mono-methoxylated flavonoids, while flavedo was richer in poly-methoxylated flavonoids and hydroxylated fatty acids. Moreover, further research is required to more accurately evaluate the effectiveness, the toxicity, and the mechanism of action of many PMFs as well as to increase their bioavailability by using readily accessible and appropriate drug delivery vehicles. Further analysis of more orange samples from other sources has yet to be evaluated to obtain a bigger picture of the entire respective population of orange samples. In addition, other factors, i.e., seasonal variation, storage conditions and agricultural practices may be assessed using the same platform.

## Figures and Tables

**Table 1:** Source of the different *Citrus sinensis* (var. Valencia) specimens used in the analysis.

Code		Orange Part	Country
AS	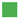	Albedo peel	Spain
AU	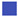	Albedo peel	Uruguay
CB	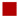	Juice concentrate	Brazil
FS	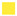	Flavedo peel	Spain
FU	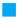	Flavedo peel	Uruguay
JS	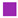	Juice	Spain
JU	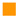	Juice	Uruguay

## Data Availability

Not applicable.

## Supplementary Materials

The following supporting information can be downloaded at: https://www.mdpi.com/article/10.3390/foods12030579/s1, Figure S1: MS^2^ Spectra of A: ferulic acid hexoside [M−H]^−^ 355.1024, C_16_H_19_O_9_^−^, B: sinapic acid pentoside [M−H]^−^ 355.1025, C_16_H_19_O_9_^−^. Figure S2: MS^2^ spectra of citropten [M−H]^−^ 205.0495, C_11_H_9_O_4_^−^. Figure S3: MS^2^ spectra of naringenin-*O*-hexosyldeoxyhexoside [M−H]^−^ 579. 1687 C_27_H_31_O_14_^−^. Figure S4: MS^2^ spectra of apigenin-*C*-hexoside-*O*-pentoside [M−H]^−^ 563.1416, C_26_H_27_O_14_^−^. Figure S5: MS^2^ spectra of hesperidin [M−H]^−^ 609.1832, C_28_H_33_O_15_^−^. Figure S6: MS^2^ spectra of deacetylnomilin [M + H]^+^ 473.222, C_26_H_33_O_8_^+^. Figure S7: MS-based OPLS of flavedo from Uruguay (FU) against that from Spain (FS) (*n* = 3) negative ionization (**A**) score plot and (**B**) relevant loading S-plot; positive ionization (**C**) score plot and (D) relevant loading S-plot. Figure S8: MS-based OPLS of albedo from Uruguay (AU) against that from Spain (AS) (*n* = 3) negative ionization (**A**) score plot and (**B**) relevant loading S-plot; positive ionization (**C**) score plot and (**D**) relevant loading S-plot. Figure S9: MS-based OPLS of orange juice from Uruguay (JU) against that from Spain (JS) (*n* = 3) negative ionization (**A**) score plot and (**B**) relevant loading S-plot. Figure S10: MS-based OPLS of orange juice from Uruguay (JU) and Spain (JS) and orange concentrate (CB) (*n* = 3) positive ionization (**A**) score plot and (**B**) relevant loading plot. References [63,64] are cited in the supplementary materials.
