# Supplementary material for: Comparative Untargeted Metabolic Profiling of Different Parts of Citrus sinensis Fruits via Liquid Chromatography–Mass Spectrometry Coupled with Multivariate Data Analyses to Unravel Authenticity
Source: Foods. 2023 Jan 29;12(3):579. doi: 10.3390/foods12030579 (PMC9914239; doi:10.3390/foods12030579)
Supplement: Supplementary file 1 [file foods-12-00579-s001.zip › foods-2147729-supplementary.pdf]

Article

Comparative untargeted metabolic profiling of different parts of *Citrus sinensis* fruits via liquid chromatography-mass spectrometry coupled with multivariate data analyses to unravel authenticity

Sherif M. Afifi[1,2*], Eman M. Kabbash[3], Ralf G. Berger[4], Ulrich Krings[4] and Tuba Esatbeyoglu[2*]

[1] Pharmacognosy Department, Faculty of Pharmacy, University of Sadat City, Sadat City 32897, Egypt; shshsh38@hotmail.com
[2] Institute of Food Science and Human Nutrition, Department of Food Development and Food Quality, Gottfried Wilhelm Leibniz University Hannover, Am Kleinen Felde 30, 30167 Hannover, Germany; esatbeyoglu@lw.uni-hannover.de
[3] National Organization for Drug Control and Research, Phytochemistry department, Giza (P.B. 12622), Egypt; emmy_700@hotmail.com
[4] Institute of Food Chemistry, Gottfried Wilhelm Leibniz University Hannover, Callinstraße 5, 30167 Hannover, Germany; rg.berger@lci.uni-hannover.de (R.G.B.), krings@lci.uni-hannover.de (U.K.)
* Correspondence: sherif.afifi@fop.usc.edu.eg (S.M.A.), esatbeyoglu@lw.uni-hannover.de (T.E.)

3.1.5. Identification of fatty acids and fatty acid amides

In the second half of the chromatogram, a considerable number of fatty acids were detected (**Figure 1**). The ESI–MS spectra revealed the presence of fourteen fatty acid derivatives preferentially ionized in the negative mode. Two trihydroxylated fatty acids were identified as peaks **36** [$(M-H)^-$ *m/z* 327.2175 $(C_{18}H_{31}O_5)^-$], and **39** [$(M-H)^-$ *m/z* 329.2333 $(C_{18}H_{33}O_5)^-$], assigned as trihydroxy-octadecadienoic acid, and trihydroxyoctadecenoic acid, respectively. Peak **59** [$(M-H)^-$ *m/z* 311.2225 $(C_{18}H_{31}O_4)^-$] showed a dihydroxylated fatty acid identified as dihydroxy-octadecadienoic acid previously detected in *C. reticulata* and *C. aurantiifolia* peels [1]. Likewise, several monohydroxylated fatty acids were detected in peaks **31** [$(M-H)^-$ *m/z* 303.2171 $(C_{16}H_{31}O_5)^-$], **50** [$(M-H)^-$ *m/z* 253.1443 $(C_{18}H_{15}O_7)^-$], **62** [$(M-H)^-$ *m/z* 293.2115 $(C_{18}H_{29}O_3)^-$], and **63** [$(M-H)^-$ *m/z* 295.2276 $(C_{18}H_{31}O_3)^-$], assigned as hydroxyhexadecanedioic acid, γ-lactone hydroxy-dodecenedioic acid methyl ester, hydroxyl-linolenic acid and hydroxylinoleic acid, respectively. The conspicuously high abundance of hydroxyfatty acids in the flavedo peel from Uruguay (FU) might result from autoxidation because of its direct exposure to atmospheric oxygen. These compounds are of interest due to their reported anti-inflammatory, cytotoxic and antimicrobial activities [2].

Fatty acid amides are natural self-defense agents in plants with a broad spectrum of supposed bioactivities, such as anti-inflammatory, anti-diabetic and antimicrobial effects [3]. They were previously detected in cold-pressed and distilled essential oils of *Citrus* species [3]. In positive mode analysis they undergo amide bond cleavage with neutral loss of the fatty acid to yield a fragment ion at [$M+H-17$ $(NH_3)]^+$. Two fatty acid amides were identified

including peak **65** [$(M+H)^+$ *m/z* 338.3428 $(C_{22}H_{44}NO)^+$] with a MS^2 fragment ion at *m/z* 321 [M+H−17 $(NH_3)]^+$ identified as erucamide (docosenamide); peak **38** [$(M+H)^+$ *m/z* 280.2677 $(C_{18}H_{34}NO)^+$] was identified as linoleamide, detected in all parts of *Citrus sinensis* from Spain, but absent in samples from Uruguay. Thus, the compound may serve as an indicator to distinguish between *Citrus sinensis* from both suppliers. They were detected previously in the peel of *Citrus paradisi* and *Citrus grandis* [3].

3.1.6. Identification of nitrogenous compounds

Positive ionization mode succeeded in the identification of a number of nitrogen containing compounds. Peak **27** showed a $(M+H)^+$ at *m/z* 249.0614 $(C_8H_{14}N_2O_5P)^+$ with fragment ions at *m/z* 169 [M+H−80 $(HPO_3)]^+$ and 81 $[(H_2PO_3)]^+$, was identified as pyridoxamine phosphate, a vitamin B6 phosphate. It was identified in all analyzed samples being most prominent in juice concentrate from Brazil. Peak **37** [$(M+H)^+$ *m/z* 316.2827 $(C_{18}H_{38}NO_3)^+$] was identified as hydroxy-sphingenine, an unsaturated ceramide previously identified in *Citrus unshiu* [4]. It was detected for first time in all analyzed samples being most prominent in juice concentrate from Brazil (CB). Peak **60** [$(M-H)^-$ *m/z* 194.0821 $(C_{10}H_{12}NO_3)^-$] was identified as *N*-phenylacetylglycine and found most prominent in CB and JS.

Suppl. Fig. S1. MS² Spectra of A: Ferulic acid hexoside [M−H]⁻ 355.1024, $C_{16}H_{19}O_9^-$, B: Sinapic acid pentoside [M−H]⁻ 355.1025, $C_{16}H_{19}O_9^-$

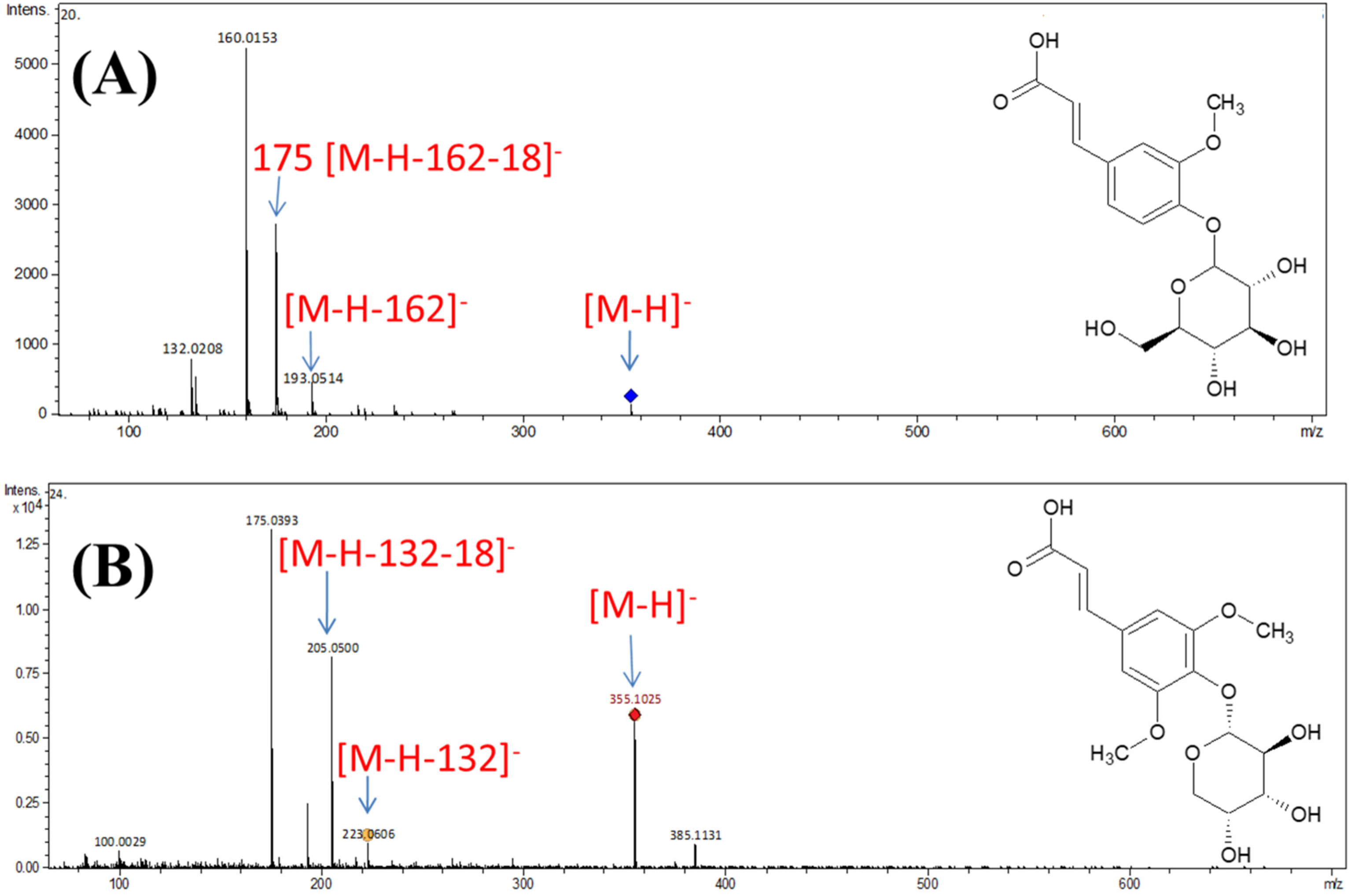

Suppl. Fig. S2. MS² Spectra of citropten [M−H]$^-$ 205.0495, $C_{11}H_9O_4^-$

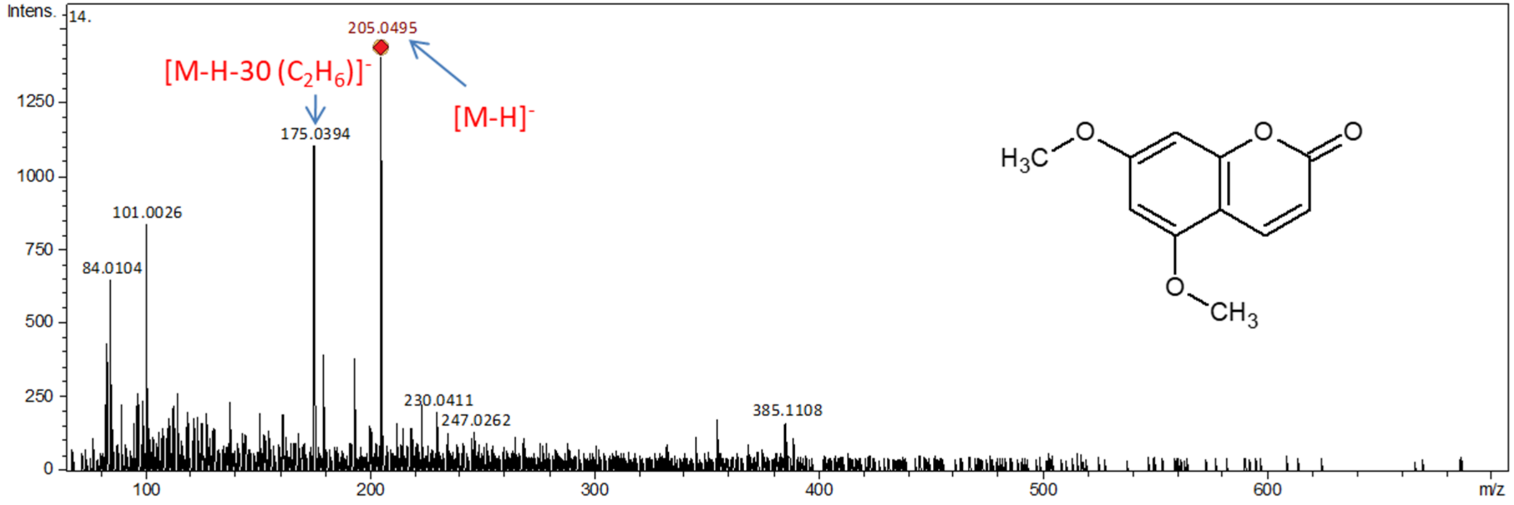

Suppl. Fig. S3. MS² Spectra of naringenin-*O*-hexosyldeoxyhexoside [M−H]$^-$ 579. 1687 $C_{27}H_{31}O_{14}^-$

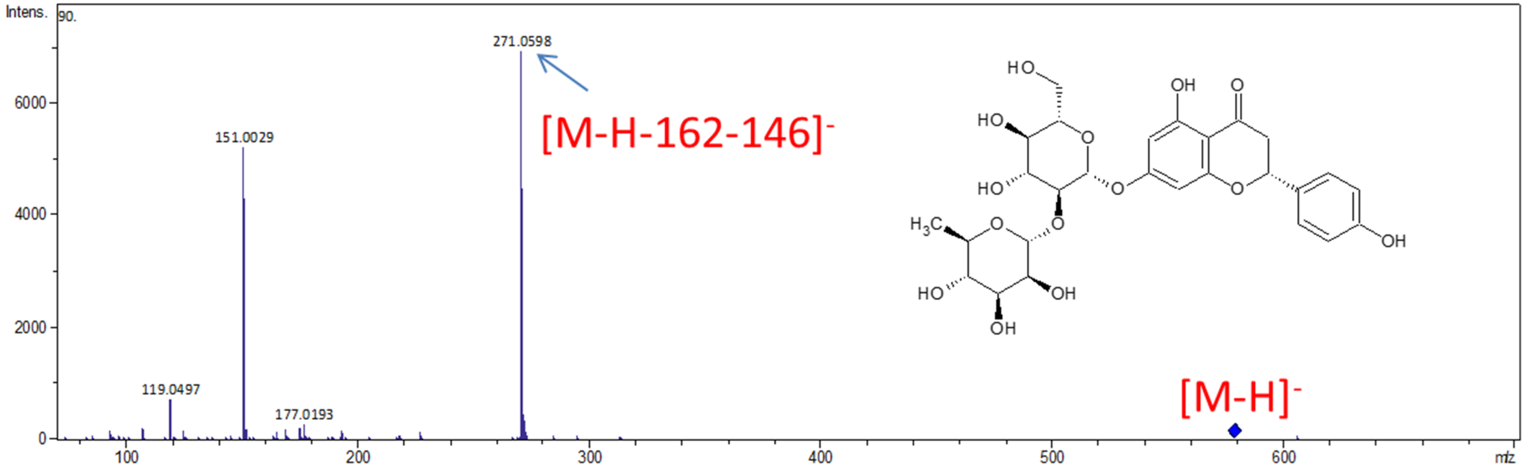

Suppl. Fig. S4. MS² Spectra of apigenin-*C*-hexoside-*O*-pentoside [M−H]⁻ 563.1416, $C_{26}H_{27}O_{14}^{-}$

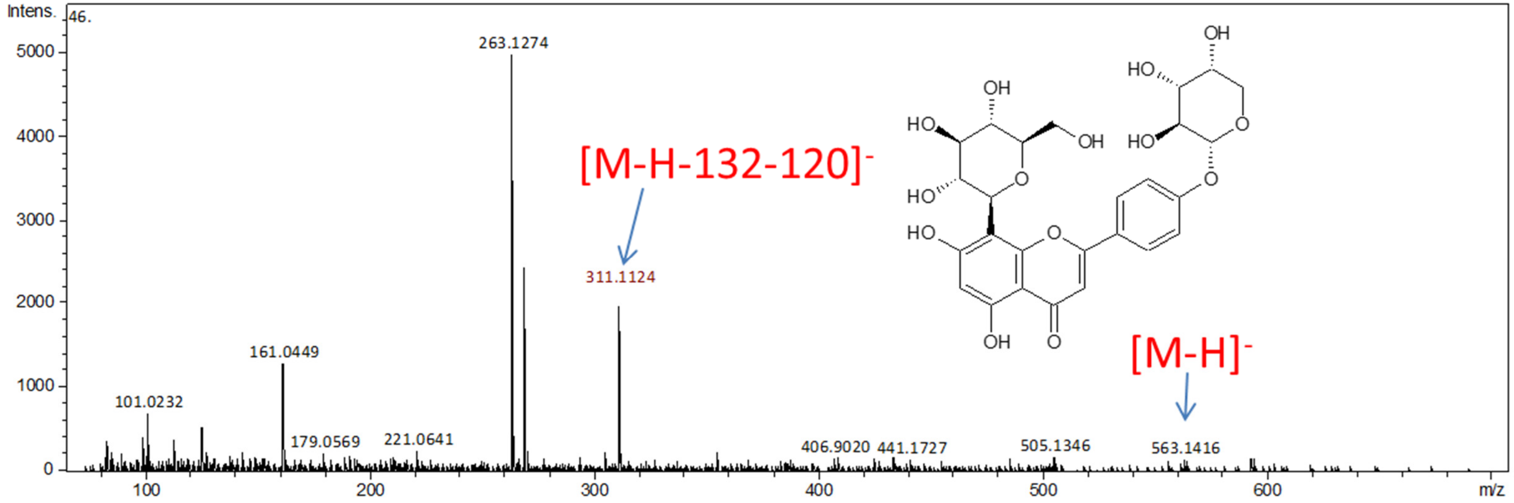

Suppl. Fig. S5. MS² Spectra of hesperidin [M−H]⁻ 609.1832, $C_{28}H_{33}O_{15}^{-}$

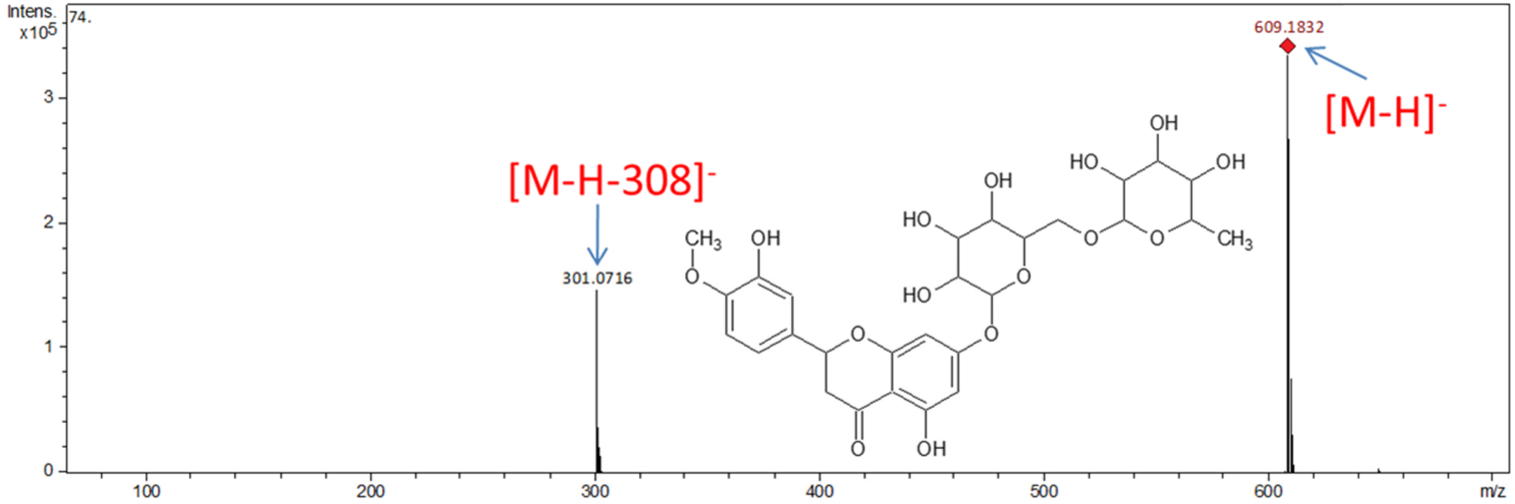

Suppl. Fig. S6. MS^2 Spectra of deacetylnomilin $[M+H]^+$ 473.222, $C_{26}H_{33}O_8^+$

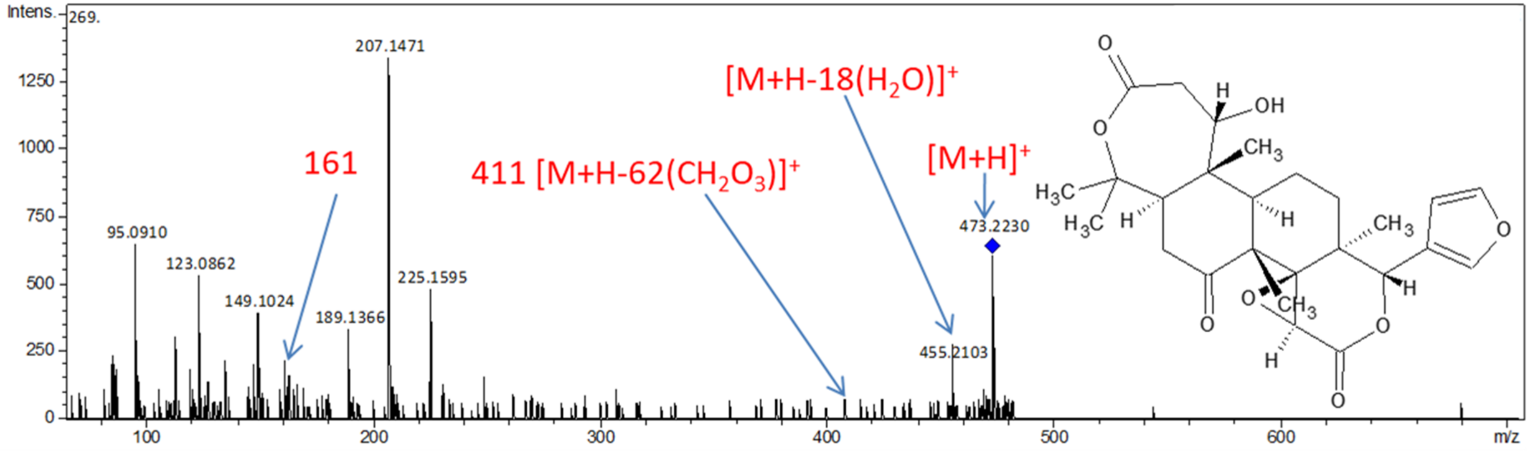

Suppl. Fig. S7. MS based OPLS of flavedo from Uruguay (FU) against that from Spain (FS) (n=3) negative ionization (A) score plot and (B) relevant loading S-plot; positive ionization (C) score plot and (D) relevant loading S-plot.

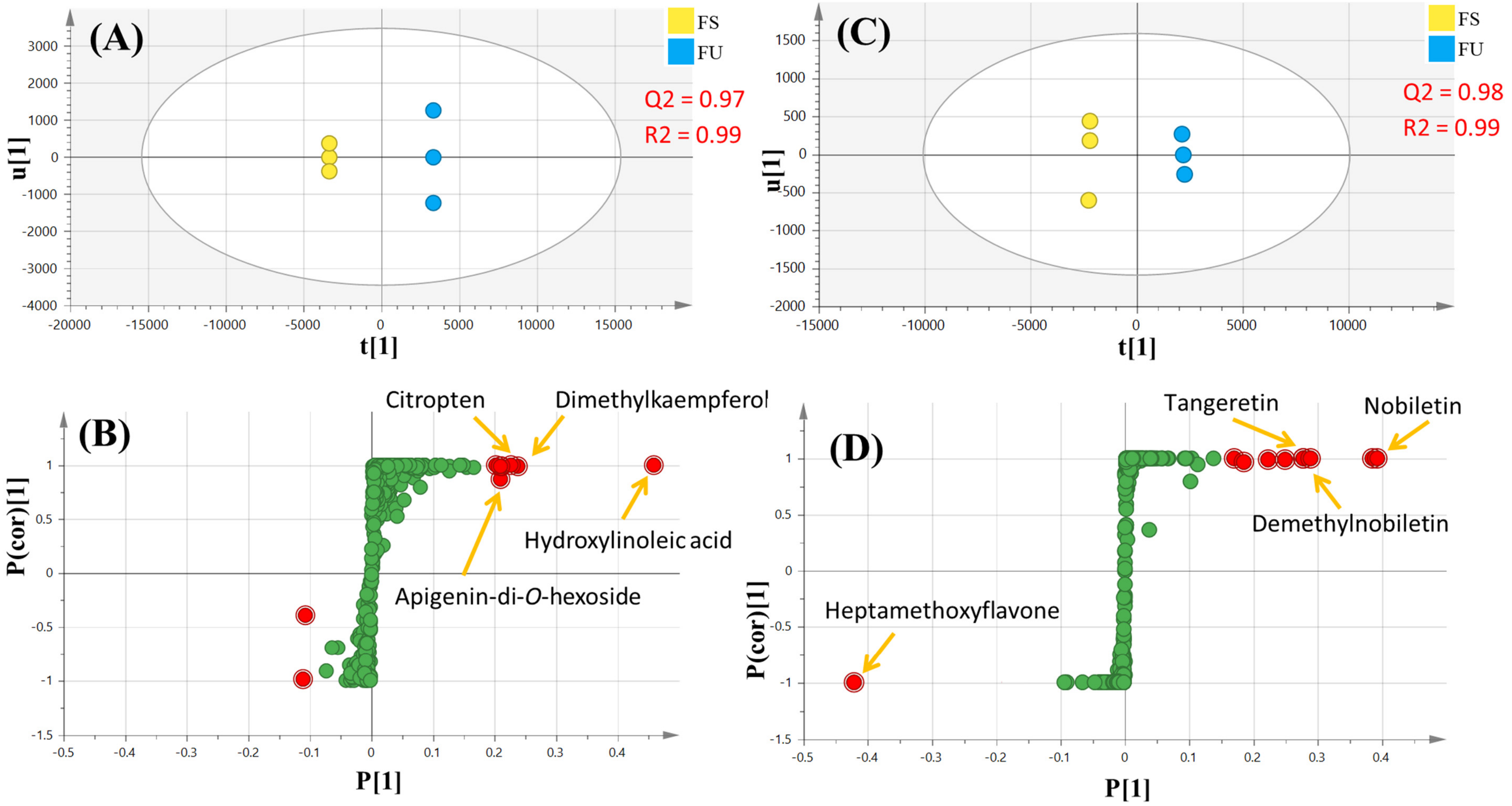

Suppl. Fig. S8. MS based OPLS of albedo from Uruguay (AU) against that from Spain (AS) (n=3) negative ionization (A) score plot and (B) relevant loading S-plot; positive ionization (C) score plot and (D) relevant loading S-plot.

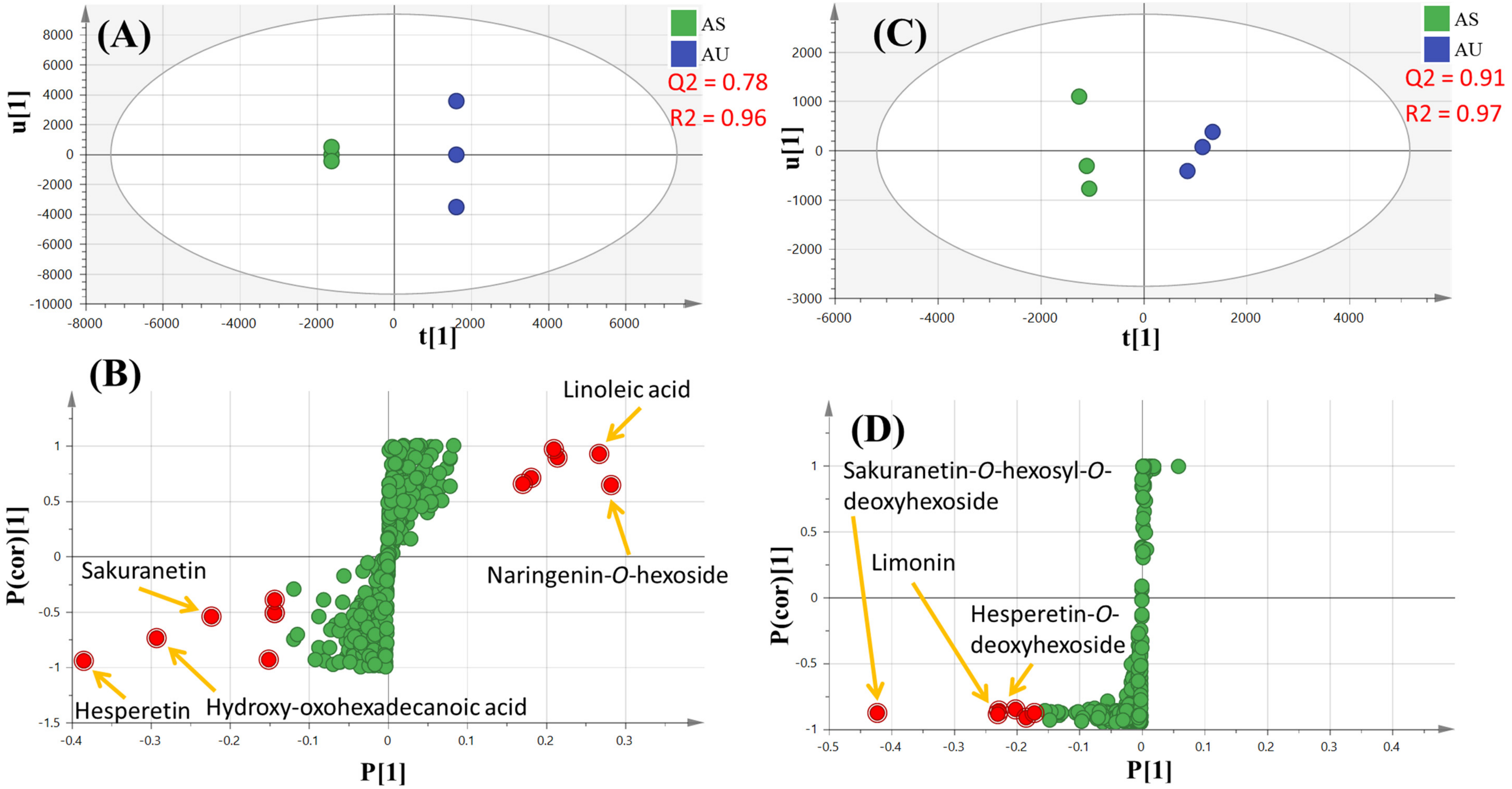

Suppl. Fig. S9. MS based OPLS of orange juice from Uruguay (JU) against that from Spain (JS) (n=3) negative ionization (A) score plot and (B) relevant loading S-plot.

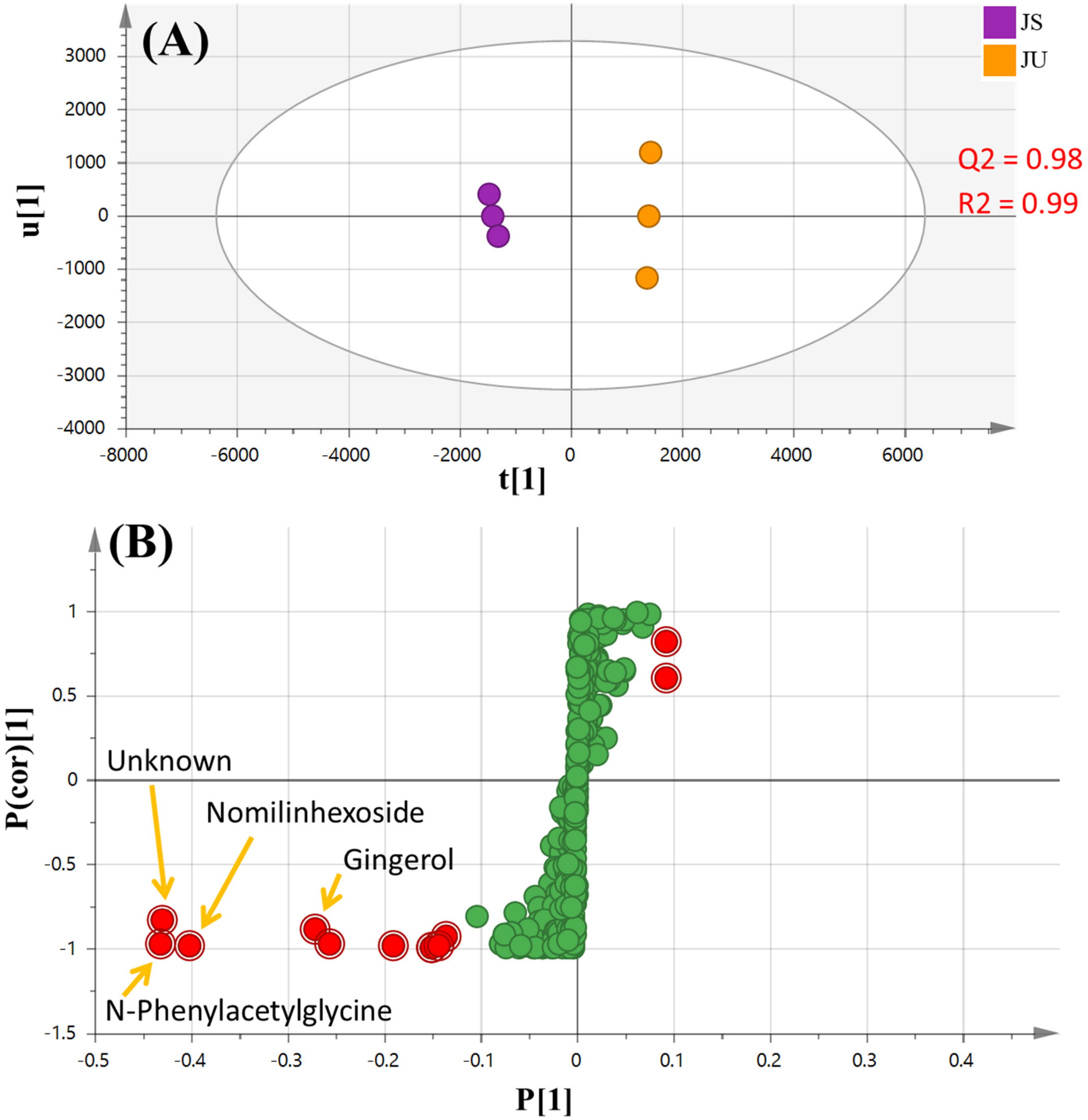

Suppl. Fig. S10. MS based OPLS of orange juice from Uruguay (JU) and Spain (JS) and orange concentrate (CB) (n=3) positive ionization (A) score plot and (B) relevant loading plot.

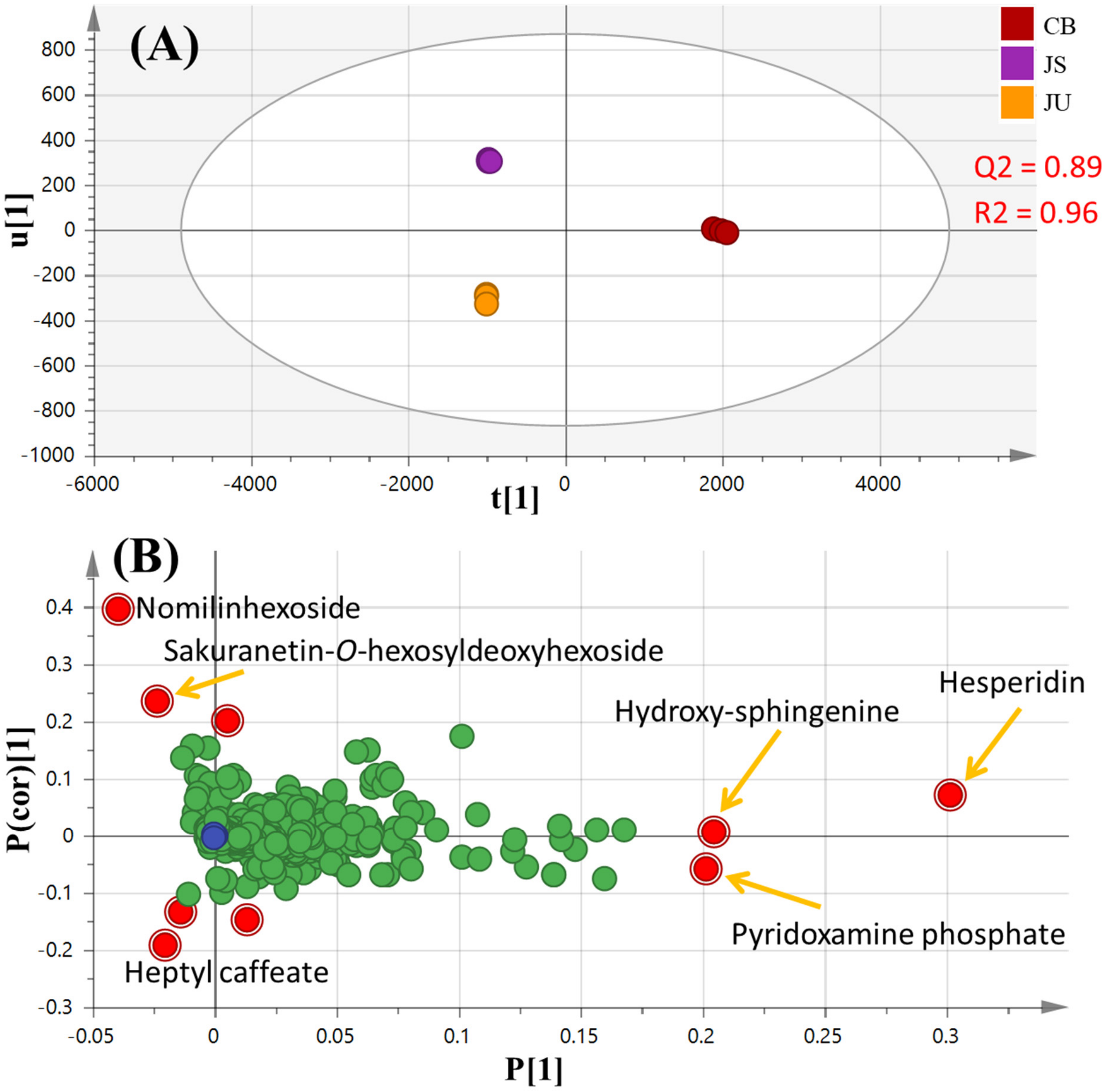

98
